# The G Protein-Coupled Estrogen Receptor (GPER) Is Expressed in Two Different Subcellular Localizations Reflecting Distinct Tumor Properties in Breast Cancer

**DOI:** 10.1371/journal.pone.0083296

**Published:** 2014-01-08

**Authors:** Eleftherios P. Samartzis, Aurelia Noske, Alexander Meisel, Zsuzsanna Varga, Daniel Fink, Patrick Imesch

**Affiliations:** 1 Department of Gynecology, University Hospital Zurich, Zurich, Switzerland; 2 Institute of Surgical Pathology, University Hospital Zurich, Zurich, Switzerland; 3 Department of Medical Oncology, University Hospital Zurich, Zurich, Switzerland; Wayne State University School of Medicine, United States of America

## Abstract

**Introduction:**

The G protein-coupled estrogen receptor (GPER) is a novel estrogen receptor that mediates proliferative effects induced by estrogen but also by tamoxifen. The aim of our study was to analyze the frequency of GPER in a large collective of primary invasive breast carcinomas, with special emphasis on the subcellular expression and to evaluate the association with clinicopathological parameters and patient overall survival.

**Methods:**

The tissue microarrays from formalin-fixed, paraffin embedded samples of primary invasive breast carcinomas (n = 981) were analyzed for GPER expression using immunohistochemistry. Expression data were compared to the clinicopathological parameters and overall survival. GPER localization was also analyzed in two immortalized breast cancer cell lines T47D and MCF7 by confocal immunofluorescence microscopy.

**Results:**

A predominantly cytoplasmic GPER expression was found in 189 carcinomas (19.3%), whereas a predominantly nuclear expression was observed in 529 cases (53.9%). A simultaneous comparable positive expression of both patterns was found in 32 of 981 cases (3.2%), and negative staining was detected in 295 cases (30%). Confocal microscopy confirmed the occurrence of cytoplasmic and nuclear GPER expression in T47D and MCF7. Cytoplasmic GPER expression was significantly associated with non-ductal histologic subtypes, low tumor stage, better histologic differentiation, as well as Luminal A and B subtypes. In contrast, nuclear GPER expression was significantly associated with poorly differentiated carcinomas and the triple-negative subtype. In univariate analysis, cytoplasmic GPER expression was associated with better overall survival (p = 0.012).

**Conclusion:**

Our data suggest that predominantly cytoplasmic and/or nuclear GPER expression are two distinct immunohistochemical patterns in breast carcinomas and may reflect different biological features, reason why these patterns should be clearly distinguished in histological evaluations. Prospective studies will be needed to assess whether the expression status of GPER in breast carcinomas should be routinely observed by clinicians, for instance, before implementing endocrine breast cancer treatment.

## Introduction

The G protein-coupled estrogen receptor (GPER), formerly also known as G protein receptor 30 (GPR30), was identified as a novel estrogen receptor that mediates a rapid, non-genomic response to estrogens [1]. Interestingly tamoxifen and fulvestrant are also important known activating GPER ligands [1]. Although tamoxifen and fulvestrant are used therapeutically to inhibit the 17beta-estradiol signaling pathway in breast cancer, it has been shown in an immortalized human breast cancer cell line (MCF7) that these drugs lead to an agonistic activation of GPER that results in stimulated proliferation via EGFR transactivation [2]. Therefore, GPER has also been experimentally showed to mediate the proliferative effects of tamoxifen in the endometrium [3]. Supporting these findings, GPER expression has been clinically correlated with tamoxifen-induced endometrial thickening and bleeding [4].

Previous studies in breast cancer patients reported an association of GPER expression with an increased metastatic potential and a poorer prognosis [5]. GPER may also play an important role in developing tamoxifen resistance in breast cancer, because GPER activation leads to a suppression of the TGF-beta signaling, which is supposed to be an important mechanism in this process [6]. However, in breast cancer cells that were negative for the classical estrogen receptors, it has also been shown that estrogen or hydroxytamoxifen were able to induce cell proliferation and migration via an activation of GPER, which seems to be mainly mediated by the connective tissue growth factor (CTGF) [7].

This mechanism is of great clinical relevance because it indicates that tamoxifen may have a cancer-promoting effect through GPER, which raises the question whether GPER expression should be assessed routinely in breast cancer patients. This question is supported by the results of a study that reported on significantly reduced survival in patients with initially GPER-positive tumors who were treated with tamoxifen compared to GPER-negative tumors, which suggests that patients with a high GPER expression should not be treated with tamoxifen alone [8].

In the literature, GPER has been reported to be expressed in approximately 60% of all breast carcinomas [1]. However, data about the expression frequency and subcellular expression pattern of GPER in breast carcinomas are based on a rather limited number of immunohistochemical studies [5], [8], [9]. Whereas only the cytoplasmic GPER expression was detected in two of these studies [5], [9], breast cancer specimens showing a cytoplasmic and nuclear staining were described in another study [8].

The aim of our study was to investigate the GPER expression rate and pattern in a large collective of breast carcinomas, with special emphasis on the subcellular GPER expression pattern in correlation to relevant clinicopathological factors and patient overall survival.

## Materials and Methods

### Patients

The study was approved by the local ethics committee (ref. number StV-Nr. 12-2005; Kantonale Ethikkommission Zürich, Stampfenbachstrasse 121, 8090 Zürich, Switzerland). The local ethics committee waived the need for written informed consent from the participants for this retrospective tissue microarray study. Tissue microarrays from formalin-fixed, paraffin-embedded samples of primary invasive breast carcinomas were constructed as previously described [10]. The carcinomas were diagnosed at the Institute of Surgical Pathology (University Hospital Zurich, Switzerland) between 1991 and 2005. Tumor tissue samples from 981 patients (female n = 976 and male n = 5) were suitable for investigation. The histological type was based on the 2003 WHO classification. Tumor grading was performed according to Bloom and Richardson [11], as modified by Elston and Ellis [12]. The hormone receptor expression, Her2 status as well as MIB1 (Ki-67) proliferation index (cut-point 10%) were previously analyzed [10], [13]. All of the carcinomas were classified according to the so-called intrinsic subtypes, such as Luminal A and B, HER2 positive and triple-negative [14].

### Immunohistochemistry

Tissue microarray sections were processed using the Ventana Benchmark automated staining system (Ventana, Tuscon, AZ, USA). For the antigen retrieval, the slides were incubated with cc1 buffer (cell conditioning solution cc1; tris-based buffer with slightly alkaline pH 6) for 10 min. Staining was performed with a rabbit polyclonal anti-GPCR (GPR30, GPER) antibody (Abcam, ab39742, dilution 1∶50). The specificity of this antibody has been verified in two independent studies [15], [16]. We used the same antibody for another study, which therefore served as a positive control [17]. Normal breast tissues (n = 52) were included on the TMAs and served as an internal positive control. Negative controls by omission of the primary antibody were included. An evaluation of the immunohistochemical staining was performed by two authors (AN, AM).

### Immunofluorescence microscopy

The three used immortalized breast carcinoma cell lines (MCF7, T47D, and MDA-MB231) were cultured using standard methods in DMEM supplemented by 10% fetal bovine serum and 1% antibiotic/antimycotic substance in an incubator at 37°C in an atmosphere of 5% CO2 and 95% humidity. MDA-MB231 is a GPER-negative cell line [18] and was included as a negative control. The dishes were subcultured when 90% confluence was attained. For the immunofluorescence experiments, subcultured cells were directly seeded on microscopy coverslips and allowed to attach for 24 h in the culture dish. The coverslips were then fixated in 4% paraformaldehyde, permeabilized with PBS 0.2% triton x-100 and blocked in bovine serum albumin 5%. The same primary rabbit polyclonal anti-GPCR (GPR30, GPER) antibody (Abcam, ab39742, dilution 1∶100) as for the immunohistochemistry was used for these experiments. Incubation with the primary antibody was done at 4°C overnight. After washing 3-times in PBS the coverslips were incubated in the secondary anti-rabbit antibody Alexa Fluor 488 (Invitrogen, dilution 1∶1000) for 30 min, washed 3-times in PBS, mounted in DAPI staining and transferred on microscope slides. The complete experiments were performed two times independently and each of them in duplicates. Conventional (Leica DMI6000B) and confocal (Leica SP5, using the hybrid detection system Leica HyD) immunofluorescence microscopy was performed. 3-dimensional analysis of the confocal images was done using Imaris software (version 7.6.4, Bitplane, Zurich, Switzerland).

### Statistics

The statistical analysis was performed using the IBM SPSS version 20 (SPSS Inc., Chicago, IL, USA). The GPER expression data were dichotomized according to the median in negative and positive groups. Fisher's exact test was used to assess the statistical significance of the associations between the GPER expression and clinicopathological features. The univariate survival analysis was performed using Kaplan-Meier method, survival curves were compared with the log-rank test. Additionally, univariate and multivariate COX regression analyses were carried out. Only cases with clinical follow-up data (n = 782) were considered for the survival analysis. P-values<0.05 were considered as significant.

## Results

### Expression of GPER in invasive breast carcinomas

**Figure 1:**
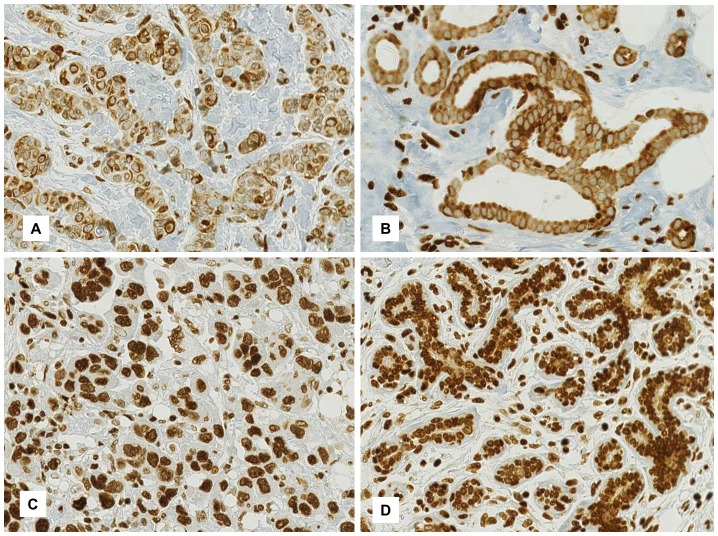
Immunohistochemical expression of GPER in invasive breast carcinoma. A: A predominantly cytoplasmic expression in a moderate differentiated invasive ductal carcinoma. B: A predominantly cytoplasmic expression in a well differentiated invasive ductal carcinoma with a perinuclear accentuation. C: Strong nuclear expression in a poorly differentiated invasive ductal breast cancer. D: Epithelium of terminal ductal-lobular units of normal breast tissue shows strong nuclear expression. The magnification of all images is 200×.

The GPER immunohistochemistry revealed two distinct expression patterns: predominantly nuclear and/or predominantly cytoplasmic. Therefore, both patterns were evaluated separately for each sample. Representative images of the staining pattern are indicated in Figure 1. In total, 981 primary invasive carcinomas were investigated for GPER expression. Cytoplasmic expression was found in 189 cases (19.3%), whereas nuclear expression was observed in 529 cases (53.9%). Simultaneously, the positive expression of both patterns was found in 32 of 981 cases (3.2%) and negative staining was detected in 295 cases (30%). The repartition in either the cytoplasmic or the nuclear GPER expression pattern was significant for the breast cancer samples (p<0.0001). Nuclear but no cytoplasmic staining was observed in the luminal and myoepithelial cells of all the normal breast tissue samples (n = 52).

### Localization of GPER in breast cancer cell lines

**Figure 2:**
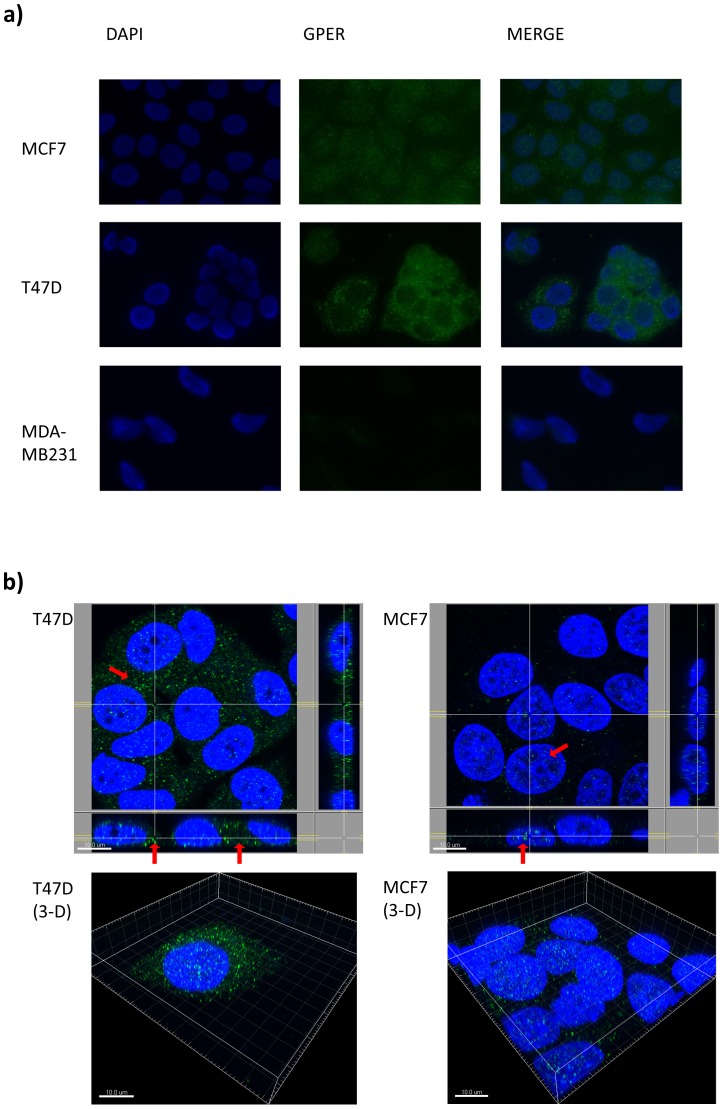
Analysis of GPER localization by conventional and confocal immunofluorescence microscopy. Representative images of two independent experiments each performed in duplicates. A: Immunofluorescence microscopy showing a different expression pattern in MCF7 (predominantly nuclear) and T47D (predominantly cytoplasmic). The GPER-negative MDA-MB231 cell line was used as negative control. B: Confocal microscopy in T47D and MCF7 using a Leica SP5 microscope (with Leica HyD hybrid detection system). T47D show a strong GPER expression which is mainly localized in the cytoplasm of the cell. No distinct membranous expression was observed. MCF7 show a less strong GPER expression, which is clearly detectable inside the nucleus by analysis of the confocal images.

The breast carcinoma cell lines T47D and MCF7 showed different GPER expression patterns in the immunofluorescence experiments (Figure 2a). We observed a strong GPER expression in T47D which was mainly localized in the cytoplasm. In contrast, MCF7 which in comparison expressed GPER less strongly showed a mainly nuclear localization of GPER. Both localizations were confirmed by confocal microscopy as shown in Figure 2b.

### Association of GPER with clinicopathological factors

The clinicopathological characteristics of the breast carcinomas are shown in Table 1. Follow-up data were available in 782 of the cases. The median follow-up period was 47 months (range 0 to 394 months). The adjuvant therapy data were unavailable.

**Table 1:** Clinicopathological parameters of the primary invasive breast carcinomas (n = 981).

Characteristics	n	%
Age at diagnosis		
<60 years	383	39.0
≥60 years	418	42.6
missing	180	18.3
Histologic subtype		
ductal	777	79.2
lobular	139	14.2
others	63	6.4
missing	2	0.2
Tumor stage		
pT1	393	40.1
pT2–4	585	59.6
missing	3	0.3
Nodal stage		
pN0	364	37.1
pN1–3	489	49.8
unknown	128	13.0
Histologic grade		
G1	150	15.3
G2	471	48.0
G3	345	35.2
unknown	15	1.5
Subtypes*		
Luminal A	216	22.0
Luminal B (HER2 −)	432	44.0
Luminal B (HER2 +)	70	7.1
HER2	51	5.2
Triple negative	112	11.4
unknown	100	10.2

The carcinomas were classified according to the so-called intrinsic subtypes [13].

To evaluate an association of GPER expression in breast cancer with clinicopathological parameters, we performed a statistical analysis as given in Table 2. We observed that cytoplasmic GPER expression was significantly associated with histologic subtypes other than invasive-ductal, low tumor stage (pT1), well and moderate histologic grade, and Luminal A and B “intrinsic subtypes”.

**Table 2:** Correlation between the clinicopathological factors and GPER expression.

Clinicopathological characteristics		GPER cytoplasmic			GPER nuclear		
		negative	positive	p	negative	positive	p
Total n = 981		792 (80.7%)	189 (19.3%)		452 (46.1%)	529 (53.9%)	
**Age at diagnosis (n = 801)**				0.979			0.747
<60 years (n = 383)		314 (82%)	69 (18%)		167 (44%)	216 (56%)	
≥60 years (n = 418)		343 (82%)	75 (18%)		187 (45%)	231 (55%)	
**Histologic subtype (n = 979)**				**0.005**			0.062
ductal (n = 777)		638 (82%)	139 (18%)		351 (45%)	426 (55%)	
lobular (n = 139)		110 (79%)	29 (21%)		63 (45%)	76 (55%)	
others (n = 63)		42 (67%)	21 (33%)		38 (60%)	25 (40%)	
**Tumor stage (n = 978)**				**0.020**			0.711
pT1 (n = 393)		303 (77%)	90 (23%)		178 (45%)	215 (55%)	
pT2–4 (n = 585)		486 (83%)	99 (17%)		272 (46%)	313 (54%)	
**Nodal stage (n = 853)**					0.801		0.541
pN0 (n = 364)		290 (80%)	74 (20%)		165 (45%)	199 (55%)	
pN1–3 (n = 489)		393 (80%)	96 (20%)		232 (47%)	257 (53%)	
**Histologic grade (n = 966)**				**<0.0001**			**0.005**
G1 (n = 150)		101 (67%)	49 (33%)		73 (49%)	77 (51%)	
G2 (n = 471)		366 (78%)	105 (22%)		240 (51%)	231 (49%)	
G3 (n = 345)		314 (91%)	31 (9%)		133 (39%)	212 (61%)	
**Subtypes** * **(n = 881)**				**<0.0001**			**<0.0001**
Luminal A (n = 216)		160 (74%)	56 (26%)		107 (50%)	109 (50%)	
Luminal B (HER2−) (n = 432)		330 (76%)	102 (24%)		214 (50%)	218 (50%)	
Luminal B (HER2+) (n = 70)		58 (83%)	12 (17%)		33 (47%)	37 (53%)	
HER2 (n = 51)		51 (100%)	0		22 (43%)	29 (57%)	
Triple negative (n = 112)		109 (97%)	3 (3%)		35 (31%)	77 (69%)	
**ER (n = 933)**				**<0.0001**			0.003
negative (n = 167)		164 (98%)	3 (2%)		59 (35%)	108 (65%)	
positive (n = 766)		589 (77%)	177 (23%)		369 (48%)	397 (52%)	
**PR (n = 720)**				**<0.0001**			0.007
negative (n = 247)		221 (89%)	26 (11%)		99 (40%)	148 (60%)	
positive (n = 473)		368 (78%)	105 (22%)		240 (51%)	233 (49%)	
**HER2 (n = 935)**				**0.006**			0.965
negative (n = 812)		641 (79%)	171 (21%)		378 (47%)	434 (53%)	
positive (n = 123)		110 (89%)	13 (11%)		57 (46%)	66 (54%)	

Intrinsic subtypes [13].

In contrast, nuclear GPER expression was significantly associated with a higher histologic grade (poorly differentiated carcinomas) and triple-negative “intrinsic subtype”.

### Association of GPER with overall survival

**Figure 3:**
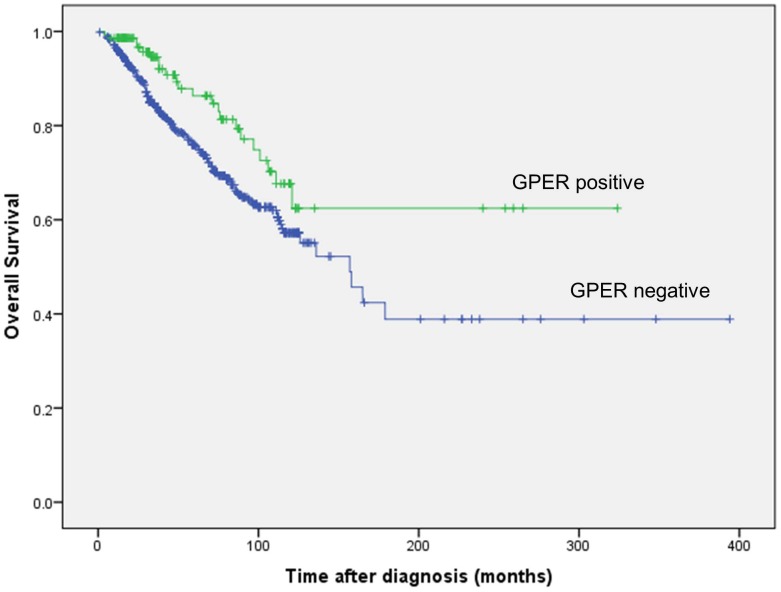
Kaplan-Meier analysis: Better overall survival in patients with positive cytoplasmic GPER expression compared to negative cytoplasmic GPER expression (log rank, p = 0.012).

In the univariate survival analysis, positive cytoplasmic GPER expression was associated with better overall survival (log rank, p = 0.012), as shown in Figure 3. In a multivariate analysis, adjusted for other prognostic clinicopathological factors like patient age, tumor and nodal stage, histologic grade and so-called intrinsic subtypes (as shown in Table 3), the prognostic significance of cytoplasmic GPER could not be confirmed. Nuclear GPER expression did not show any correlation with overall survival.

**Table 3:** Univariate COX regression analysis: factors predicting overall survival.

Clinicopathological characteristics	n	Overall survival
		HR (CI), p-value
Age at diagnosis	782	1.7 (1.3–2.3), 0.0001
<60 years	374	
≥60 years	408	
Tumor stage	779	3.1 (2.2–4.4), 0.0001
pT1	317	
pT2–4	462	
Nodal stage	672	2.6 (1.8–3.8), 0.0001
pN0	275	
pN1–3	397	
Histologic grade	770	1.6 (1.2–2.0), 0.0001
G1	109	
G2	376	
G3	285	
Subtypes*	881	1.1 (1.0–1.3), 0.011
Luminal A	216	
Luminal B (HER2 negative)	432	
Luminal B (HER2 positive)	70	
HER2	51	
Triple negative	112	
GPER	782	0.6 (0.4–0.9), 0.013
cytoplasmic −	641	
cytoplasmic +	141	
GPER	782	0.9 (0.7–1.3), 0.69
nuclear −	347	
nuclear +	435	
GPER	782	0.9 (0.8–1.), 0.14
cytoplasmic −/ nuclear −	233	
cytoplasmic −/ nuclear +	114	
cytoplasmic +/ nuclear −	408	
cytoplasmic +/ nuclear +	27	

Intrinsic subtypes [13].

## Discussion

Our study provides the immunohistochemical staining results of GPER in the largest cohort of 981 primary breast carcinomas to date. We observed GPER expression (predominantly cytoplasmic and/or nuclear) in 70% of the studied breast carcinomas. GPER expression was distinguishable between the cytoplasmic (19.3% of the carcinomas) and nuclear (53.9%) compartment. The majority of the tumor specimen showed either nuclear or cytoplasmic staining, whereas only 3.2% of the tumors showed simultaneous nuclear and cytoplasmic staining. No distinct membranous staining was detectable neither by immunohistochemistry in the tissue microarray nor by immunofluorescence in the immortalized breast cancer cell lines MCF7 and T47D.

The rate of cytoplasmic GPER expression, however, was markedly lower in our study compared with two previous reports that observed cytoplasmic expression in approximately 60% of the breast carcinoma cases [5], [8]. Although this may most likely be the result of a different patient collective and number of cases, it has of course also to be noted that different antibodies against GPER were used in each of the two mentioned studies as well as in our study.

The expression pattern of GPER and its subcellular localization is still a subject of debate. Because these specimens were placed on the same tissue microarray, it is not likely that the distribution of cytoplasmic and nuclear GPER staining that was observed in our study was simply the result of an artifact. Moreover, the specificity of the same antibody used in our study has been verified in an independent study using shRNA for GPER (negative control) as well as the specific inducer G1 (positive control) [15] and it has been shown in a second study using the western blot technique that this antibody does not bind to ER-alpha [16]. Our immunofluorescence experiments confirm the specificity of the antibody and different GPER localization depending on the cell line (predominantly cytoplasmic localization in T47D and mainly nuclear localization in MCF7). Confocal microscopy evidenced the occurrence of both, cytoplasmic and nuclear localizations in these cell lines.

The distinct histopathological occurrence of cytoplasmic and nuclear GPER expression observed in our study may most likely be explained by studies that have investigated the dynamical changes of the subcellular localization of GPER [19], [20]. It has been shown by cellular surface labeling that a retrograde transport of GPER from the plasma membrane towards the nucleus occurs with a consecutive accumulation of GPER in the perinuclear space followed by a later dispersion in the cytoplasm [20]. Additionally, in another recent study it has been shown that estradiol can stimulate nuclear translocation of GPER in breast cancer-associated fibroblasts, indicating that GPER also mediates a nuclear signaling pathway [21], [22]. Although the biological meaning of subcellular GPER trafficking has not been definitively clarified, it may be the result of a functional receptor modulation [20], which is of major importance because it could possibly implicate a different biological response to GPER signaling in different breast carcinomas. The observed different staining pattern may therefore be the reflection of a dynamic time-dependent intracellular GPER trafficking process, which nevertheless may be differently modulated according to the biological characteristics of different breast carcinoma subtypes.

Our results showed that cytoplasmic GPER expression was associated with low tumor stage and well- to moderately differentiated carcinomas. Moreover, cytoplasmic GPER expression was significantly associated with hormone receptor-positive breast carcinoma subtypes Luminal A and B. These results are in line with the results of Ignatov et al., which showed a tendency to associate cytoplasmic GPER positivity with ER and PR positive breast carcinomas [8]. Filardo et al. described a significant association of cytoplasmic GPER positivity to ER positivity but did not observe an association with PR expression [5]. Most likely because of the larger number of analyzed invasive breast carcinoma samples in our study compared with these studies, we were clearly able to observe a significant correlation between cytoplasmic GPER positivity and ER- and PR-positive breast carcinoma samples.

In contrast to the cytoplasmic GPER expression, we observed that nuclear GPER expression was associated with poorly differentiated carcinomas and a triple-negative intrinsic subtype. This opposite association of cytoplasmic and nuclear-localized GPER with clinicopathological parameters might be the reflection of a different biological significance of the two different subcellular GPER localizations [20].

Despite recent data [5], [8], [9], [23], the clinical relevance of GPER in breast cancer remains relatively poorly investigated. GPER expression in breast cancer is of clinical relevance because it has been shown that GPER may trigger a proliferative response to estrogen in cases of ER-alpha and ER-beta negative but GPER-positive breast cancers [7]. GPER may also be implicated in the processes of decreased sensitivity or resistance to tamoxifen in ER-positive and GPER-positive breast cancer because tamoxifen is known to cause a GPER-mediated proliferative effect in breast cancer cells [6], [7]. Furthermore, GPER is most likely involved in the endometrial proliferation that is frequently observed in tamoxifen treatment [3], [4].

In their study of 323 patients and a confirmation cohort of 103 patients, Ignatov et al. found an association between increased GPER expression and a shorter RFS in patients undergoing tamoxifen therapy. Conversely, in patients who were not subjected to a tamoxifen therapy, GPER was associated with a longer RFS. In addition, the authors were able to demonstrate in 33 paired biopsies (before and after adjuvant therapy) that GPER expression significantly increased only under tamoxifen treatment [8].

One limitation of our study was the fact that the clinical data on systemic therapy were not available. Consequently, we were unable to verify the relationship between GPER expression and resistance to anti-hormonal drugs in our collective. Our data provide a purely descriptive approach to the relationship between GPER expression status and different tumor characteristics. Further studies are warranted to provide more mechanistic data and information about possible GPER protein modifications which were not subject of this study. Even if the subcellular localizations of the detected GPER protein were confirmed by confocal microscopy, it is not excluded that other forms of GPER were not detectable and it is not proven that all the detected forms were reflecting a fully functional protein. Nevertheless, our data is clearly indicating a differential subcellular GPER expression between different invasive breast carcinoma tissue and cell lines, which is associated with different clinicopathological characteristics and should be taken into consideration in further studies.

### Conclusion

In conclusion, our data suggest that cytoplasmic and nuclear GPER expression are two relatively distinct immunohistochemical patterns in breast carcinomas and may reflect different biological features; therefore, these patterns should be clearly distinguished in histological evaluations. Our findings provided a systematic analysis of the GPER expression pattern in a large number of breast carcinomas, which indicated that cytoplasmic GPER expression in breast carcinomas is generally associated with a better clinical outcome, whereas a nuclear GPER expression is associated with less favorable tumor properties. The intracellular trafficking of GPER has been shown in vitro [19], [20] and may reflect distinct biological behavior of the tumors, which must be further investigated in future studies. Prospective studies will be needed to assess whether the expression status of GPER in breast carcinomas should be routinely observed by clinicians, for instance, before implementing breast cancer treatment with tamoxifen. Nevertheless, it appears to be of importance to distinguish between distinct subcellular localizations when assessing the GPER expression pattern immunohistochemically in breast carcinomas, which will also be relevant for upcoming studies in this field.
